# Aspiration of Subdural Hygroma Using Augmented Reality Neuronavigation: A Case Report

**DOI:** 10.7759/cureus.69070

**Published:** 2024-09-10

**Authors:** Andrew Janssen, Yinghua Jiang, Aaron S Dumont, Pervez Khan

**Affiliations:** 1 Neurosurgery, Tulane University School of Medicine, New Orleans, USA

**Keywords:** augmented reality, hygroma, image guidance, mixed reality, neurocritical care, virtual reality

## Abstract

Augmented reality (AR) is emerging as a key technology in neurosurgery. Projecting three-dimensional (3D) anatomic models onto the surgical field provides unique operative information to make procedures safer and more efficient. A small footprint, rapid registration AR system was used for bedside guidance during aspiration of a subdural hygroma. A 77-year-old male presented for resection of a suprasellar tumor and subsequently developed a large bilateral subdural hygroma. We performed the aspiration of the hygroma at the bedside using AR guidance. The AR system allowed for precise needle placement during the aspiration. The aim of this report was to demonstrate the clinical feasibility of integrating a novel AR system into the clinical workflow of a bedside procedure. As AR continues to expand in the field, the benefits of this technology for various procedures will become more evident.

## Introduction

A subdural hygroma is a medical condition characterized by the accumulation of fluid between the dura mater, the outermost layer of the meninges, and the arachnoid membrane, which covers the brain. Unlike a subdural hematoma, which involves blood accumulation, a subdural hygroma consists of a clear, colorless fluid [1,2]. This condition often arises following head trauma, surgery, or spontaneously due to other medical issues, and can lead to increased pressure on the brain, causing symptoms such as headaches, confusion, or neurological deficits. Early diagnosis and management are crucial to prevent complications and ensure optimal recovery [1-3]. Traditionally, the evacuation of a subdural hygroma is performed using standard surgical techniques in the operating room, without the use of navigation systems.

Recent advances in medical technology have introduced augmented reality (AR) as a promising tool in neurosurgery [4-6]. AR involves projecting digital information onto the real-world environment, providing surgeons with enhanced visualization and real-time guidance during procedures. For drainage of a subdural hygroma, AR can be used to superimpose imaging data directly onto the patient's head, allowing for precise localization of the fluid collection and surrounding critical structures. The device described uniquely utilizes a small 1.2 lb headset, making bedside neuronavigation for specific indications feasible. This innovative approach has the potential to improve operative accuracy, reduce procedural time, and enhance patient outcomes.

Here, we describe the use of AR to assist in the bedside draining of a subdural hygroma. The aim of this report was to demonstrate the clinical feasibility of integrating a novel AR system into the clinical workflow of a bedside procedure. We discuss the advantages of the small footprint and rapid AR system and how it allowed us to perform the procedure successfully at the bedside versus the operating room. 

## Case presentation

A 77-year-old male visiting from China who was diagnosed with a pituitary tumor in 2017 presented to us with increased lethargy, confusion, episodes of nausea and vomiting, and a witnessed episode of loss of consciousness. The CT head scan demonstrated a multilobulated sellar/suprasellar mass. The patient underwent sublabial transsphenoidal resection, followed by a second staged pterional craniotomy during the same admission.

**Figure 1:**
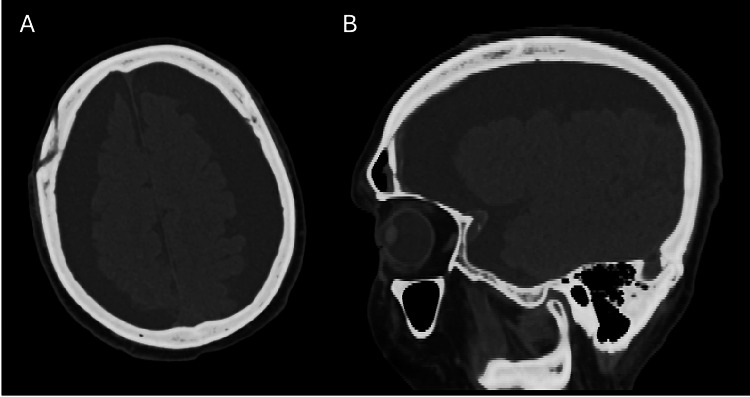
(A) Axial and (B) sagittal preoperative CT imaging displaying the bilateral subdural hygroma

Postoperative imaging showed a decreased size of the tumor compared to the previous exam.  During the postoperative hospital course, the patient unfortunately experienced an episode of aspiration pneumonia and septic shock and underwent subsequent intubation. After weeks of medical management, the patient was successfully extubated. The patient remained somnolent, however, opening his eyes only to verbal and painful stimuli and was found on CT imaging to have a large bilateral subdural hygroma (Figure 1).

Surgical drainage was chosen to further optimize the patient’s mental status. The traditional evacuation approach usually involves bringing the patient back to the OR with intubation and general anesthesia. The patient was at very high risk of requiring tracheostomy and ventilation dependence, which would significantly increase the flight cost and feasibility of returning home to China. After discussing the risks and benefits with the patient's family, we planned to use a headset-based AR system to perform a bedside subdural hygroma evacuation through the previous craniotomy without intubation or general anesthesia. In particular, the 3D reconstruction included the burr holes and burr hole covers from the previous craniotomy. We therefore were able to see the virtual location of the covers beneath the patient's skin in order to guide the large bore needle for pressure evaluation and aspiration. The procedure went very well; the AR system rapidly and accurately registered the 3D model of the patient's cranial anatomy from his CT imaging and successfully displayed a virtual trajectory toward the targeted subdural area that was subsequently used to position the drainage catheter during insertion.  

During aspiration, it was noted that the overall intracranial pressure was low, and after evacuation of 50+ cc fluid, the pressure zeroed out and it was deemed safe to stop, confirming that the hygromas were not significantly contributing to the patient's altered mental status. The patient was further managed in the neuro ICU and his mentation and neurological status continued to improve over the next week.

AR guidance system 

Segmenting Anatomical Structure 

The CT scan was used to generate a 3D model of the patient’s anatomy. The following structures were manually segmented using ITK Snap: skull, brain, intradural fluid collection, and burr hole covers. The segmentations and resulting 3D models were confirmed as acceptable by the proceduralists prior to using the AR headset.

AR Technology Platform 

The entirety of the AR system (Hoth Intelligence, Philadelphia, Pennsylvania, United States) operates out of the Microsoft Hololens 2 headset (Microsoft Corporation, Redmond, Washington, United States) [7,8]. The headset is a wireless head-mounted display that superimposes digital 3D models within the user's real-world view via an optical see-through display.

3D Model Registration to the Patient 

**Figure 2:**
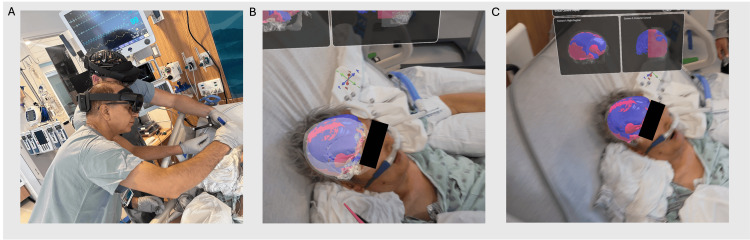
Surgeon's view through the headset during presurgical planning: Bedside use of the augmented reality system (A) Microsoft Hololens 2 headsets were worn to display anatomical model; (B,C) Surgeon’s view through the headset showing patient-specific 3D anatomy registered onto the head—brain (pink), fluid (blue) and skull (white).

**Figure 3:**
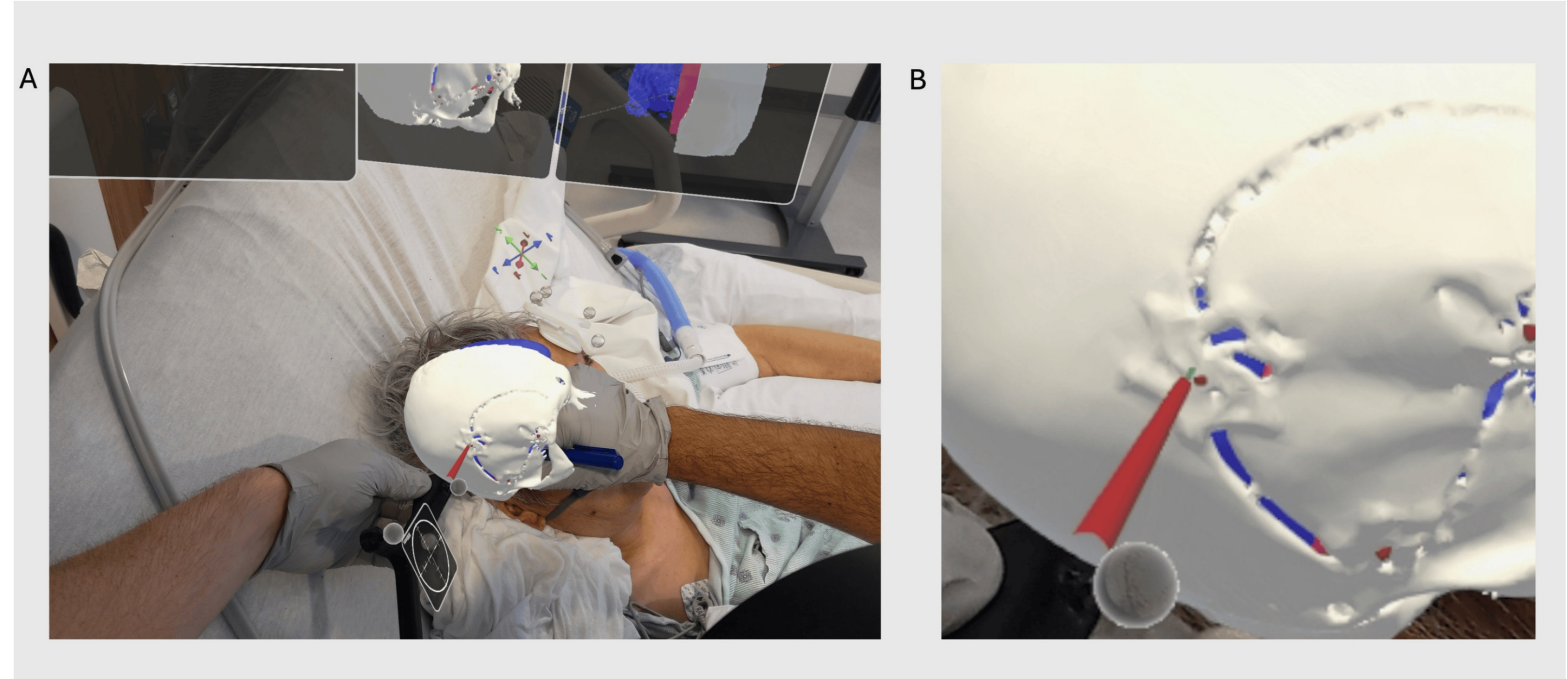
Surgeon's view through the headset during aspiration of subdural hygroma: Planning needle insertion using augmented reality (A) Three-dimensional (3D) skull model overlaid onto patients’ head. A custom-tracked stylet was used to pinpoint the preexisting burr hole cover.  (B) Magnified view of the overlaid 3D model of the burr hole cover which served as the target for needle placement.

The novel AR system used in this case allows the surgeon to visualize the patient's 3D anatomic anatomy registered and overlaid onto the patient’s head during the procedure. Prior to making an incision, the surgeon has visibility of the skull, burr hole covers, and the location of the fluid surrounding the brain (Figure 2). As a bedside solution, this system satisfies the key requirements of being small, rapid, and fiducial-less. The advantage of the system is its size, ease of use, and its rapid, fiducial-less registration process. The system comprises a single headset (and thus has excellent mobility in the ICU), registration is approximately 20 seconds, and any imaging protocol can be used since the system does not require fiducial markers. The registration process leverages the sensors on the headset to capture surface features from the patient's face which are then aligned to the corresponding points from the medical scan. Once registered, the application performs spatial mapping of the environment which allows the surgeon to move freely around the patient’s head and visualize the 3D anatomy from different perspectives. Figure 3 shows the surgeon planning the needle insertion during aspiration with the help of AR.

## Discussion

Image guidance and advanced visualization are the standard of care for cranial neurosurgical procedures. Despite this, there are several neurosurgical procedures that are still performed without image guidance which can typically be attributed to cost and inconvenience. These systems are expensive and require large immobile equipment which is less conducive for an ICU setting. AR, while once reserved for educational purposes, has seen tremendous growth in the field of neurosurgery, with a growing number of reports demonstrating the intraoperative use of this technology [4-6]. We describe a novel and practical use of bedside AR neuronavigation during the drainage of a subdural hygroma. 

At our institution, this procedure is typically performed in the operating room. Normally, image guidance is not available in the bedside setting. The AR system enabled us to safely and quickly perform this procedure at the patient’s bedside by providing accurate visualization of the subdural target. We decided to take advantage of the Burr hole covers, including their location in the 3D model, present on the patient's head to define the insertion point of the needle used to evacuate the fluid. A challenge of this approach is precisely identifying the Burr holes and, more specifically, the small gaps between the metal burr hole hall covers which are not visible to the surgeon. The AR system clearly displayed the location and depth of volume of the fluid collection and the location of the borehole covers in the proper orientation on the patient's head. We were therefore able to accurately place the needle at the precise location in order to access the fluid collection more easily. It took a single needle stick for both needle aspirations, and altogether this led to a faster, less morbid, and more precise procedure and avoided the need to take the patient to the operating room, which was of important economic and social significance to the patient. 

The strength of this technology lies in the footprint and speed with which it operates. The system did not require a computer cart or tower and the registration workflow was completed in under a minute which fits perfectly into the clinical workflow of a bedside neurosurgical procedure. This report demonstrates a simple yet effective use of augmented reality. Additionally, by displaying the 3D anatomy overlaid onto the patient’s head, as opposed to on a separate computer monitor, we were able to maintain focus on the surgical field while leveraging the advanced visualization provided by the AR system. This clinical application demonstrates the feasibility of this system to provide image guidance at the bedside. Previous studies have demonstrated the clinical feasibility of other bedside procedures [9]. It is our expectation that this work will serve as a catalyst for other surgeons to introduce AR into the standard of care for bedside procedures. 

## Conclusions

The integration of AR into neurosurgical procedures has tremendous potential to improve surgical outcomes. By overlaying critical anatomical information and real-time imaging data onto the surgeon's field of view, AR can significantly enhance precision and safety. In this report, AR was effectively used to safely aspirate a subdural hygroma at the bedside, obviating the need to perform the procedure in the operating room. The technology facilitated more accurate needle placement and improved overall procedural efficiency. Further research and clinical trials will be essential to refine these applications and validate their benefits in diverse surgical contexts.
